# Current status of vascularized composite tissue allotransplantation

**DOI:** 10.4103/2321-3868.130184

**Published:** 2014-04-06

**Authors:** Karoline Edtinger, Xiaoyong Yang, Hanae Uehara, Stefan G. Tullius

**Affiliations:** 1 Division of Transplant Surgery and Laboratory of Transplant Surgery Research, Brigham and Women’s Hospital and Harvard Medical School, 75 Francis St, Boston, Massachusetts 02115 USA; 2 Department of Surgery, University Hospital Regensburg, University of Regensburg, Regensburg, Germany; 3 Division of Urology, Bejing Chao-Yang Hospital, Capital Medical University, Bejing, China; 4 Department of Plastic and Reconstructive Surgery, Osaka Medical College, Takatsuki, Osaka, Japan

**Keywords:** Face transplantation, hand transplantation, immunosuppression, composite tissue transplantation

## Abstract

Vascularized composite tissue allotransplantation (VCA) offers treatment options of complex functional deficiencies that cannot be repaired with conventional reconstructive methods. VCAs consist of blocks of functional units comprising different tissue types such as skin, bone, muscle, nerves, blood vessels, tendons, ligaments and others, and are thus substantially different from the composition of organ transplants. The field of VCA has made fascinating progresses in the recent past. Among other VCAs, numerous successful hand, face and limb transplants have been performed in the world. At the same time, specific questions in regard to innate and adaptive immunity, consequences of ischemia/reperfusion injury, immunosuppression, preservation, and regenerative capacity remain. In spite of this, the field is poised to make significant advances in the near future.

## Vascularized composite tissue allotransplantation (VCA) — recent developments leading to a clinical reality

Organ and tissue transplants including the replacement of limbs have been a long-standing dream.[1] At the same time, rapid advances in solid organ transplantation (SOT) paved the way for the current success of VCAs.

World War II initiated a more in-depth exploration of skin transplants for the treatment of burned warriors. These efforts had subsequently inspired a team in Boston led by Joseph E. Murray to perform the first successful SOT between identical twins.[2] The same team went on in the following years to establish successful renal transplants between nonidentical living donors, followed shortly thereafter by the first renal transplant from a deceased donor with chemical immunosuppression.[3,4]

**Table:** 

Access this article online	
**Quick Response Code**:	**Website**: www.burnstrauma.com
	**DOI**: 10.4103/2321-3868.130184

**Figure 1:**
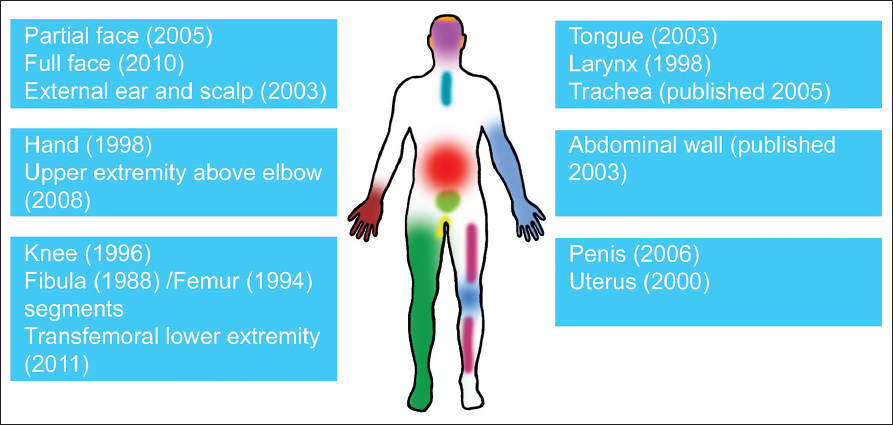
Progress in clinical vascularized composite tissue allotransplantation (VCA): Year of transplantation or publication.[12,13,15–27]

The first successful human VCA, an en block digital flexor tendon, was conducted by Erle E. Peacock Jr. in North Carolina in the absence of immunosuppression in 1957 and coined the term composite tissue allotransplantation (CTA).[5] A first hand transplant, although clinically not successful, was performed in 1964 with chemical immunosuppression in Ecuador by Robert Gilbert.[6] A long period of stagnation in VCA followed, mainly based on the paradigm that the potent antigenicity of the skin could not be sufficiently managed by immunosuppressants.[71] In the meantime, immunosuppression became more refined for SOT.[8,9] Addressing the paradigm of intensified antigenicity in VCAs, interesting experimental work in the early 1990s demonstrated that the transplantation of a whole limb was linked to a less intense immune response as compared to individual components such as the skin.[10] First successful limb transplantations in pigs, treated with a triple immunosuppressive therapy consisting of cyclosporin (CsA), mycophenolate mofetil (MMF), and prednisone encouraged a team led by Jean-Michel Dubernard in France to conduct the first successful hand transplantation in 1998.[11,12] Additional hand transplants were performed rapidly thereafter in the USA and China, while other successful composite tissue transplants such as larynx and knee transplants were subsequently reported.[13,14] First successful face transplants were performed in 2005 in France and thereafter in China and the US (Cleveland and Boston). In 2013, the transplantation of bilateral transfemoral lower extremities was reported[15] [Figure 1]. Most recently, an important regulatory issue has been resolved and VCAs are now officially covered by United Network for Organ Sharing (UNOS) guidelines since 2013 in the USA, which ended a long-lasting discussion whether allocation of VCAs should fall under the regulatory framework of tissue or organ transplants.[28]

Although major unsolved issues including the need for a more refined immunosuppression and missing long-term results remain, the last decade has seen major advances in VCA [Table 1].

**Table 1:** **Current success and challenges in VCA**

Achievements	Challenges
Excellent esthetic and functional outcome	Minimization of immunosuppression, tolerance induction
Multidisciplinary approach	Protocols for sensitized patients
Recognition as organ, regulated by OPTN	Improved monitoring
Excellent patient and graft survival	Mechanisms of rejection Acute cellular and humoral rejection
	Acute cellular and humoral rejection
	Chronic graft deterioration
	Nerve regeneration
	Definition of acceptable ischemic times
	Refined organ preservation

VCA = Vascularized composite tissue allotransplantation, OPTN = Organ Procurement and Transplantation Network

### Immunosuppression and immune modulation in VCA

The immunosuppressive regimen in VCA continues to evolve in parallel with SOT. Indeed, the refinement of long-term immunosuppression seems of particular importance for the ‘life-giving’ rather than ‘life-saving’ VCA approach. At the same time, VCAs offer opportunities for immunomodulation such as the transplantation of bony structures (including bone marrow (BM)) with potential relevance of tolerance enabling protocols and the application of topical treatment.

### Face transplantation

In face transplantation, most patients received an induction treatment with anti-thymocyte globulin (e.g., thymoglobulin) in varying doses of 1–2 mg/kg/day[16,29] for up to 10 days[29,30] after transplantation. Some teams decided on an induction treatment with an anti-interleukin-2 receptor (anti-IL-2R) antibody (e.g., basiliximab).[31,32] Steroids were administered in 500–1,000 mg intravenous (i.v.) boluses of methylprednisolone,[33,34] or, alternatively 250–500 mg prednisone[17,35] on the day of transplantation, then tapered to daily prednisone doses of 5–20 mg/day. In the VCA program at Brigham and Women’s Hospital in Boston, we were able to wean all our nonsensitized recipients off steroids.[31,36]

Maintenance therapy is usually initiated on day 1 with calcineurin inhibitors (CNIs), in general tacrolimus, in all reported face transplantations with doses adjusted to maintain through levels of 10–15 ng/ml during the first 1–3 months in majority of cases and subsequent tapering to levels as low as 3–5 ng/ml.[16,30,37] Moreover, MMF was part of the standard triple immunosuppression regimen in face transplantation and was uniformly prescribed at dosages of 2 g/day early after transplantation and tapered thereafter.[17,33] Some teams have tested the feasibility of CNI free protocols. A progressive decline in renal function or malignancies have prompted some to switch to CNI free maintenance immunosuppression based on sirolimus treatment with a targeted trough level of 8–12 ng/ml.[37,38]

Early acute rejections (grade I and II) have usually been treated with increasing dosages of maintenance immunosuppression, in some cases, topical treatment with tacrolimus and clobetazol ointments, and prednisone mouthwashes.[17,31,35] In the absence of adequate response or in more advanced rejections (grade III), steroid pulses have been necessary. A few cases required an escalation to anti-thymocyte globulin therapy plus a modification in the maintenance immunosuppression protocol.[6]

Of note, not all low grade acute rejections have required treatment and most centers have been assessing both histology and clinical presentation.[34] It remains unclear at this point, if low grade rejections are self-limiting or if the currently applied pathological scores necessarily distinguish rejection from unspecific-inflammatory events. Extracorporeal photochemotherapy (ECPCT) has been introduced in a few patients. This immunomodulatory approach demonstrated a complementary effect on preventing rejections in the absence of the known side effects of conventional immunosuppression. Although the mode of action of ECPCT is not completely clear, ECPCT has been used successfully both long-term in patients with ongoing low grade rejections and in patients with simultaneous rejections and infections.[35,39]

### Hand transplantation

Immunosuppression in hand transplantation parallels largely to regimens in other VCA approaches including face transplantation. At the same time, the experience is larger and ‘stakes’ may be slightly lower, thus allowing pushing the envelope in this area. Most teams around the world have utilized a triple immunosuppression with tacrolimus, MMF and methylprednisolone, and an induction treatment with either a polyclonal antithymocyte globulin or a monoclonal anti-IL-2R.[12] More recently, alemtuzumab (an anti-CD52 antibody) has been utilized by some centers as an induction agent.[40] Along the same line as with the experience in face transplantation, CNI-based protocols have been switched to sirolimus subsequently due to side-effects such as unstable glucose blood levels.[41,42]

### Clinical and pathological diagnosis of acute rejection in VCA

VCAs typically consist of various tissues originating from different embryological germ layers.[43] It is assumed that the skin is most sensitive to rejection, while vessels, muscles, and especially nerves are afflicted to a lesser degree.[44] Local or scattered erythema is usually considered as a clinical sign of an early acute rejection. Moderate acute rejections present with pink erythematous macules, more advanced acute rejections with scaly papules or plaques, and the irreversible grade IV state with necrosis and/or ulceration of the graft.[44]

Scoring systems based on histopathological findings classifying acute rejections of VCAs have been proposed by several groups.[45–48] In 2007, an international team of surgeons, pathologists, and basic scientists with experiences in VCA met in Banff and agreed on a standardized classification. Scores are mainly based on localization and intensity of infiltrates in the skin. Future additions to the current Banff score have been proposed and may include a graded assessment of clinical symptoms, supplemental immunohistological evaluations characterizing infiltrating cells (e.g., CD4^+^, CD8^+^ T cell or B cell) and humoral rejections.[43]

According to the Banff-07 classification, acute cellular mediated rejection is graded into 5 categories. Absent or rare infiltration define grade 0; grade I rejection is characterized by the appearance of mild perivascular infiltration. Advanced infiltrates with or without mild involvement of the epidermis, and/or adnexa defines grade II; while the addition of epithelial apoptosis, dyskeratosis, and/or keratinolysis defines grade III. Grade IV, finally, includes frank necrosis of epidermis, or other skin structures.[43]

It has been suggested that the current Banff-07 classification for VCA may not be reflective of critical aspects of rejection. In a series of more than 100 biopsies of face transplant recipients, rejections have been linked to lymphocyte associated injuries in epidermal rete ridges, follicular infundibula and dermal microvessels. Of note, during active rejection, infiltrates consisted predominantly of lymphocytes of donor origin with an immunophenotype typical of resident memory T-cell subsets. These results may not only provide additional information for a more concise grading system, but may also provide novel biological insights into the mechanisms of VCAs.[49]

### Antibody-mediated rejection (AMR)

A complex medical history including trauma and blood transfusions with subsequent sensitization is probable in patients qualifying for VCAs. In renal transplantation, patients have been transplanted against positive complement-dependent cytotoxicity (CDC) cross-matches with extensive pre- and post-transplant immunosuppression.[50] Whether similar approaches may also apply in VCA is largely unknown. VCAs have usually been followed closely for donor-recipient crossmatches comparable to the approach in SOT. Thus far, there have been only very few reports of either AMR in VCA,[51,52] circulating donor-specific antibodies (DSAs), or the deposition of the complement product C4d in VCA recipients.[52,53] Of note, AMR is currently not part of the Banff-07 VCA classification system.

Pretransplant panel reactive antibody (PRA) scores are usually used to test the degree of sensitization in solid organ and VCA recipients alike. The score provides a relative estimate with a 100% PRA predicting a positive crossmatch against any given donor. We have recently successfully treated a highly-sensitized patient who developed a fulminant AMR. This patient was treated with an intensified immunosuppression consisting of plasmapheresis, eculizumab, bortezomib and campath-1H.[54]

DSAs occurring late after hand transplantation have been reported recently. Rituximab (i.e., anti-CD20 antibody) normalized not only clinical symptoms, but also histological findings and DSA levels.[55] Another hand transplantation patient presented with an aggressive thickening of arterial walls, detected by ultrasound biomicroscopy, an advanced imaging technique capable of revealing arterial thickening noninvasively. Further tests correlated findings with the deposition of the complement product C4d. Plasmapheresis, i.v. immunoglobulin, and a switch from MMF to sirolimus inhibited further progression of the lesions.[56] Clearly, humoral immune responses are expected to gain further interest and relevance in VCA.

### Chronic graft deterioration in VCA

Although the skin is under constant surveillance, different properties of VCAs including muscle, bone, vessels, nerves, and other tissues may undergo immunologic and degenerative processes in the absence of direct visibility. The concept of ‘split rejection’, a process in which specific tissues undergo deterioration or immune attacks at a different tempo or intensity may also be of relevance impacting chronic graft deterioration in VCA.[57] As in SOT, mechanisms of chronic graft deterioration are also not well-defined in VCA. Histological and clinical findings including vascular narrowing, loss of adnexa, skin and muscle atrophy, fibrosis of deep tissue, myointimal proliferation, and nail changes indicate chronic rejection in VCA.[43]

Although confirmed, acute rejection episodes are frequent in VCA, and the field is looking back to a reasonable clinical experience, reports of chronic graft deterioration in VCA remain rare. At the same time, clinical evidence of chronic rejection exists: For example, a hand transplant recipient presented with arterial narrowing while superficial biopsies remained normal. DSAs were absent at the same time. Vasculopathy progressed, requiring subsequent amputation due to ischemia. Histological examination demonstrated predominantly vascular pathologies with hyperplasia of the intima in all donor arteries and involvement of the venous tissue as well. Of note, skin biopsies were scored grade 0-I.[56] Other patients with clinical symptoms resembling chronic changes including atrophic skin and thinning of nails, pain, cold intolerance and compromised functionality have been reported. Clinical symptoms correlated with arterial narrowing and occlusion in addition to loss of adnexa, epidermal hyperkeratosis and perivascular inflammation.[58]

Mechanisms of chronic graft deterioration remain obscure both in VCA and SOT. Hind-limb allografts in rats developed chronic changes following multiple acute rejection episodes, which were treated suboptimally. Myointimal proliferation, causing subtotal arterial occlusion was observed by day 90, while muscular atrophy and fibrosis became evident by day 60. While fibrosis has thus far been mainly linked to ischemia subsequent to advanced vasculopathy, profibrotic gene expression implicated additional causes, such as infiltrating macrophages and direct injury to muscle cells as aspects of chronic changes. Other interesting aspects include findings of mild vasculitis; while skin, muscle, and bone were unaffected, providing further evidence of the relevance of split rejection in VCA.[59]

### Clinical and experimental tolerance approaches in VCA

Tolerance protocols have been attempted in both experimental and clinical settings. The first human face transplant recipient received 2 injections of donor BM cells in consistency with the Miami protocol, however, long-term microchimerism could not be documented and triple immunosuppressive therapy plus additional immunomodulatory treatment were necessary to control rejection episodes.[37] The Pittsburgh protocol, applied in 5 hand transplantation patients, was able to keep patients on a maintenance tacrolimus monotherapy, although controls have been missing. This protocol consists of a lymphocyte-depleting induction therapy with alemtuzumab in combination with methylprednisone and a single, unmodified, donor BM cell infusion on day 14 after transplantation. DSA and C4d deposits detected in parallel to acute T cell rejections responded to topical treatment or methylprednisone infusions. Deep biopsies by 1 and 2 years revealed only focal muscle atrophy. Evaluations by high-resolution ultrasound biomicroscopy demonstrated minor to moderate vascular irregularities in 2 cases with some narrowing of luminal diameter and increased intimal-media thickness, respectively.[40] Of note, peripheral chimerism was not detected in any of the VCA patients receiving the Pittsburgh protocol.

Stable mixed chimerism, in contrast, was achieved in VCA transplants across major histocompatibility complex (MHC) barriers in large animal models. In a pig model, a non-myeloablative conditioning regimen with T cell depletion by CD3-immunotoxin, total body irradiation, and donor-specific hematopoietic stem cell transfusion (HCT) was followed by a 45 day course of CsA. VCA transplants consisting of a vascularized full thickness skin flap were performed at varying intervals after HCT. Rejections were absent during an observation period up to 500 days. Of note, 2 animals with very high chimerism levels developed graft versus host disease (GvHD). Nevertheless, GvHD could be contained with an immunosuppression consisting of CsA and steroids.[60] Achieving tolerance or the minimization of immunosuppression will be critical in improving the clinical applicability of VCAs, and future research will certainly focus on this area.

### Ischemia/reperfusion injury (IRI)

Both ischemia and reperfusion set mechanisms in place that initiate innate and adaptive immune responses contributing to acute rejection and chronic graft deterioration.[61,62] In the presence of inadequate blood supply and oxygen deprivation, imbalances of cellular ion homeostasis and an augmented permeability of cell membranes occur simultaneously.[63] Anaerobic metabolism and a buildup of lactate results in accumulation of proinflammatory mediators.[64] Of note, restored blood flow does not restore function immediately, but leads to additional damages recognized as reperfusion injury. During IRI endothelial, parenchymal and immune cells become activated and reactive oxygen species, inflammatory cytokines, and complement products contribute to a furthermore augmented injury. Additional consequences include an impaired microvascular perfusion with secondary ischemia in the transplanted organ and the promotion of a ‘cytokine storm’ that may finally lead to the development of systemic inflammatory response syndrome (SIRS).[63,65] Consequences of IRI have been extensively analyzed in SOT. IRI has only recently received attention in VCA where injury patterns of skeletal and cardial muscle demonstrated parallels.[64] As VCA is composed of different tissues, consequences of IRI may differ in a tissue-specific manner. Muscle and adipose tissue have an enhanced metabolism and are therefore more susceptible to damage.[66] Of note, viable skin could still be found in musculocutaneous autografts, although muscular tissue had already demonstrated necrotic changes.[67,68]

Time of tolerable ischemia, although of utmost clinical relevance, has thus far not been defined. In rat experiments, ischemic times exceeding 12 h drastically reduced the survival of musculocutaneous flaps. A histological analysis of biopsies in flaps with brief or prolonged ischemia (24 h) revealed no difference in the early onset of acute rejections, but demonstrated an accelerated inflammation in grafts with a prolonged ischemia.[69] Other experiments have also shown that prolonged ischemia in models of VCA-augmented adaptive immunity and increased rates of acute rejections.[70] Tolerable clinical ischemic times are currently not defined and clinical practice has aimed for brief periods of ischemia.[16,29,34,71,72] Exploring mechanisms of IRI in VCA will be critical as tolerable prolonged ischemia will increase the availability of donors. To this end, novel preservation, developed in SOT are noteworthy.[73] Pulsatile perfusion has been successfully used clinically in SOT and linked to endothelial and epithelial protection.[74–76] Future studies in VCA will need to explore if similar concepts and mechanisms may also be of relevance and benefit in VCAs.

## Conclusions

VCA has become a clinical reality during the last decade. Face and hand transplants have been successfully performed with excellent functional and esthetic outcomes by several centers in the world, and some patients have achieved a minimization of immunosuppression. While progress has been enormous, several open questions in regard to mechanisms of rejections, tolerance or minimization of immunosuppression, and the effects of unspecific injuries, consequences of IRI remain. Although critical aspects of the success of VCA are based on the experience in SOT, VCA-specific aspects need to be recognized and addressed. Most importantly, VCA is as much as SOT, a field which will thrive in a multidisciplinary approach synergizing the clinical and research expertise in tackling many unresolved issues in the field.
